# Unsuccessful Thrombin Injection for Right Femoral Artery Pseudoaneurysm: A Case Report

**DOI:** 10.7759/cureus.80640

**Published:** 2025-03-15

**Authors:** Tomohiro Nakajima, Yu Iwashiro, Tsuyoshi Shibata, Yutaka Iba, Nobuyoshi Kawaharada

**Affiliations:** 1 Cardiovascular Surgery, Sapporo Medical University, Sapporo, JPN

**Keywords:** fracture, multiple injury, pseudoaneurysm, surgical treatment failure, thrombin

## Abstract

A 33‐year‐old man was brought to the emergency department after sustaining multiple traumas. The interventional radiology team performed vascular embolization via a 6 Fr sheath inserted into the right common femoral artery due to suspected bleeding from the duodenum. The man underwent multidisciplinary management. On day 3 after injury, contrast-enhanced computed tomography (CT) revealed a pseudoaneurysm in the right common femoral artery, the radiology team opted for thrombin injection therapy, and the patient was monitored. No swelling was initially observed. However, repeat contrast-enhanced CT demonstrated a pseudoaneurysm on postinjury day 9, indicating that thrombin treatment had failed. Vascular surgery was considered, and emergency surgical intervention was performed on the same day. A pseudoaneurysm and a 6 Fr sheath puncture site were identified through a right inguinal incision, and hemostasis was achieved. The postoperative course was uneventful, and the patient was transferred for rehabilitation on postoperative day 19 following pseudoaneurysm repair. This case involved the development of a femoral artery pseudoaneurysm following endovascular treatment for multiple traumas. Although the interventional radiology team performed a local thrombin injection, the treatment ultimately failed, necessitating vascular surgery intervention. The patient underwent open surgical repair without complications. With the increasing use of local thrombin injection for pseudoaneurysms, careful post-treatment monitoring is essential, and surgical intervention should be considered at an appropriate time if necessary.

## Introduction

Pseudoaneurysms of the femoral artery are uncommon yet clinically serious complications that may arise following traumatic or iatrogenic vascular injury [1]. Ultrasound-guided thrombin injection has become a widely accepted first-line treatment for pseudoaneurysms because it is minimally invasive and highly successful [2]. However, thrombin injection may fail to achieve complete resolution, particularly in the presence of complex vascular anatomy or extensive tissue damage, thereby necessitating a transition to surgical management [3]. The early recognition of treatment failure is critical for preventing complications such as rupture or distal embolization, and emergency surgical repair offers a definitive solution in such circumstances [4]. In this report, we present the case of a 33-year-old man with a right femoral artery pseudoaneurysm in which the initial thrombin injection was unsuccessful, ultimately necessitating surgical intervention.

## Case presentation

**Figure 1:**
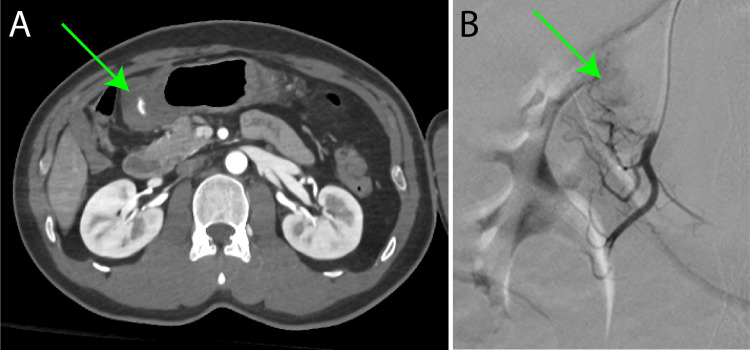
Imaging at the time of injury due to multiple traumas. (A) Contrast-enhanced computed tomography (CT) revealed active bleeding from the duodenum (green arrow). (B) Angiography confirmed the same finding (green arrow). Hemostasis was subsequently achieved using an embolic material.

A 33-year-old man presented at the emergency department after sustaining multiple traumas. The interventional radiology team performed vascular embolization using a 6 Fr sheath inserted into the right common femoral artery due to suspected bleeding from the duodenum (Figure 1). Subsequently, the patient underwent multidisciplinary treatment.

**Figure 2:**
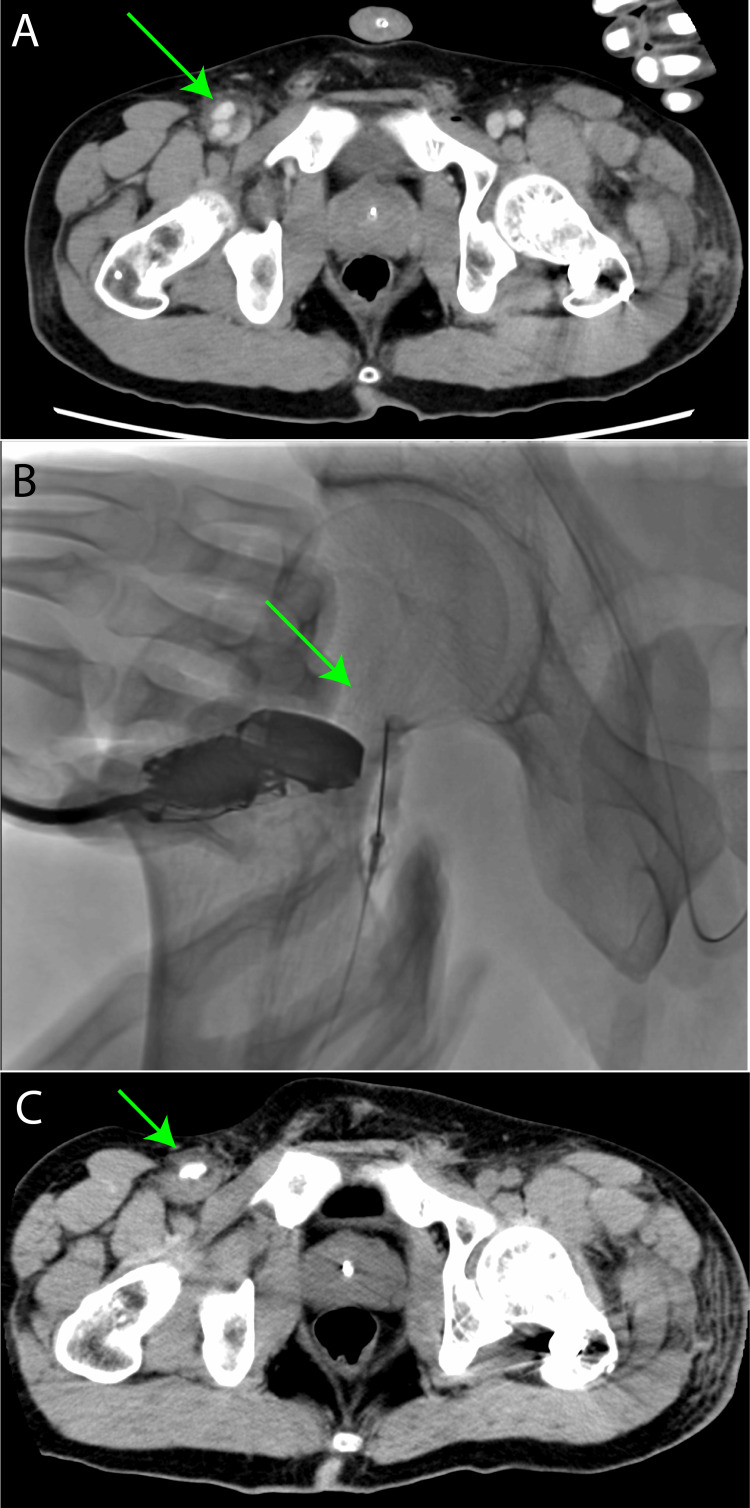
Imaging on postinjury day 3. (A) Contrast-enhanced CT showed the presence of a right femoral artery pseudoaneurysm (green arrow). (B) Thrombin was injected into the pseudoaneurysm in the angiography suite under ultrasound guidance (green arrow). (C) The contrast agent used during the procedure remained within the pseudoaneurysm (green arrow). CT: computed tomography

Contrast-enhanced computed tomography (CT) revealed a pseudoaneurysm in the right common femoral artery, and the interventional radiology team decided to proceed with thrombin injection therapy on day 3 after injury (Figure 2). The longest diameter of the pseudoaneurysm, including the false lumen, was 20 mm, and the longest diameter of the pseudoaneurysm itself was 8 mm. There was no consultation with a cardiovascular surgeon at this point. The treatment was performed by a radiation therapy physician. Under local anesthesia, an ultrasound was performed to check the blood flow entering the pseudoaneurysm. A 22 G cannula was inserted, and 2000 units of thrombin (2 mL), 3 mL of saline, and 5 mL of contrast medium were injected. An ultrasound examination five minutes after the injection confirmed that the blood flow in the pseudoaneurysm had stagnated, and the procedure was completed.

**Figure 3:**
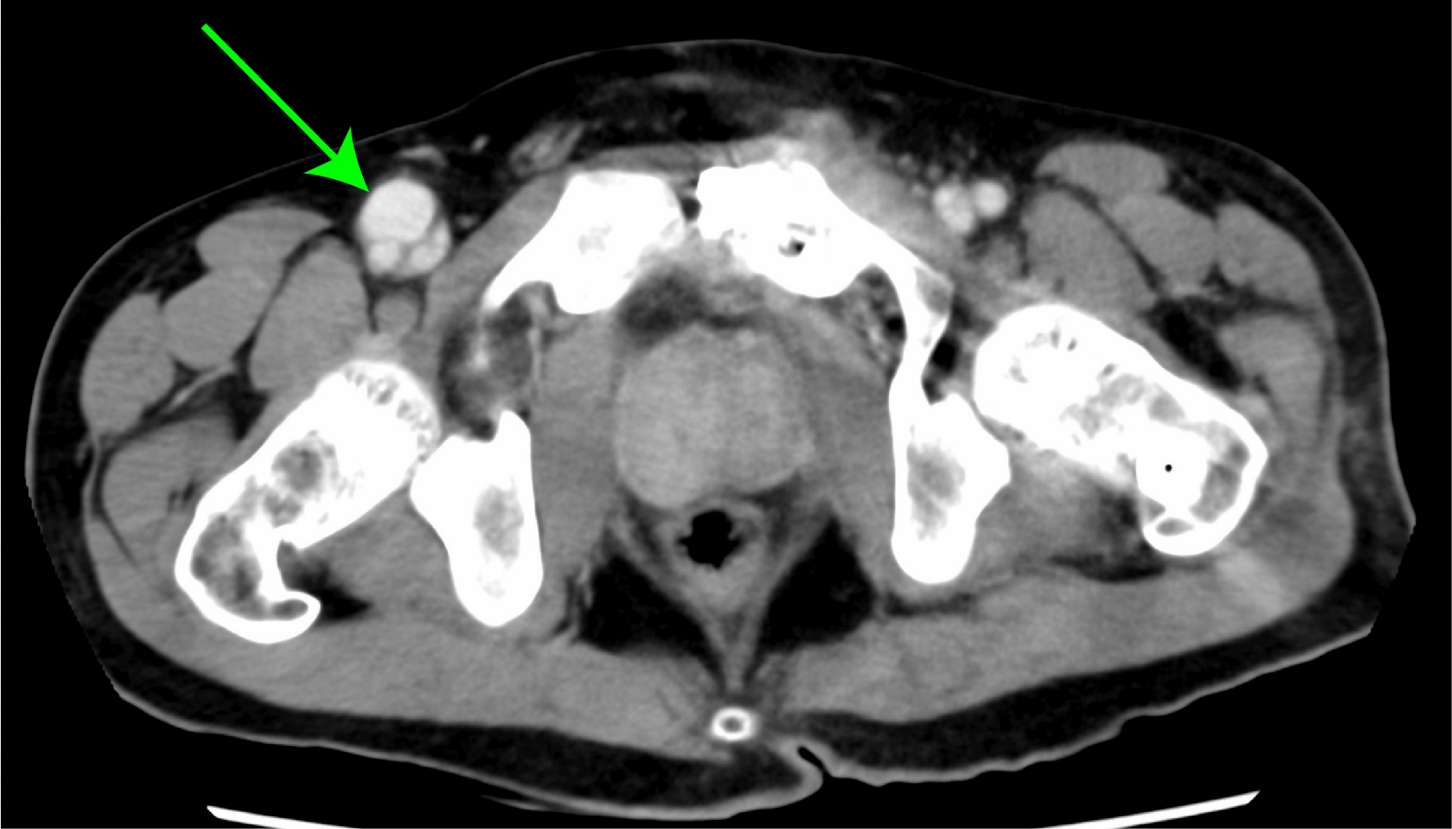
Imaging on postinjury day 14. Contrast-enhanced CT demonstrated the presence of a right femoral artery pseudoaneurysm (green arrow), which was enlarged compared with that in the previous scan. CT: computed tomography

Thrombin was administered, and the patient was monitored without any notable swelling or complications. However, pretransfer contrast-enhanced CT revealed the enlargement of the pseudoaneurysm 14 days after injury (Figure 3). The cardiovascular surgery team was consulted for the first time. Emergency surgical intervention was planned and performed on the same day given these findings.

**Figure 4:**
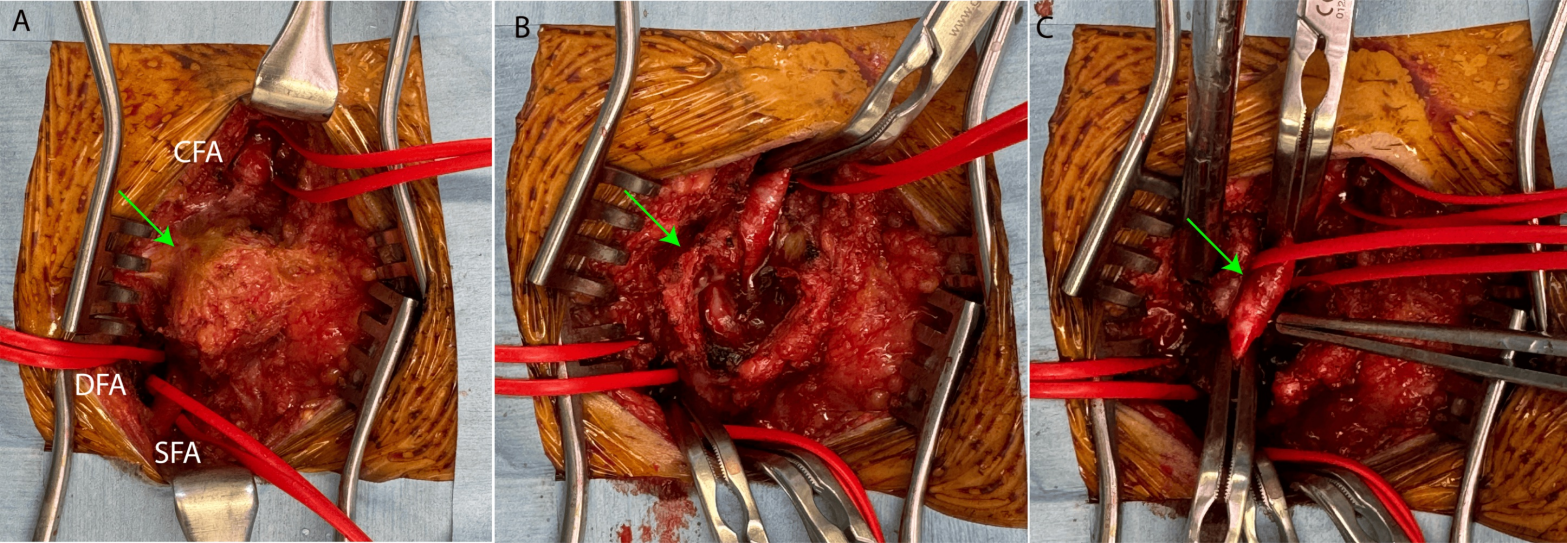
Intraoperative images. (A) A longitudinal incision was made in the right inguinal region, and the pseudoaneurysm was identified (green arrow). (B) The pseudoaneurysm was incised (green arrow). (C) The pseudoaneurysm was successfully dissected and removed. The puncture site of the 6 Fr sheath was identified (green arrow). CFA, common femoral artery; DFA, deep femoral artery; SFA, superficial femoral artery

A right inguinal incision was created, and a pseudoaneurysm was identified. Systemic heparinization was performed, and the activated clotting time (ACT) was maintained above 250 seconds, which was followed by vascular clamping to achieve hemostasis. The pseudoaneurysm was carefully dissected and separated from the surrounding normal vasculature. The puncture site of the 6 Fr sheath was identified and successfully closed using a 5-0 Prolene suture (Figure 4).

The postoperative course was uneventful, and the patient was transferred for rehabilitation on day 19 after pseudoaneurysm repair surgery. After the operation, there was no recurrence of swelling in the right groin.

## Discussion

Femoral artery pseudoaneurysms are complications following catheter-based interventions, with an incidence of up to 0.2%-7.7% depending on patient risk factors and procedural details [5]. Ultrasound-guided thrombin injection has become the preferred initial treatment because of its minimally invasive nature and its high success rate, sometimes exceeding 90% [6]. However, thrombin injection is not always successful despite its efficacy, necessitating careful post-treatment evaluation to identify the reason for treatment failure.

Several factors contribute to the failure of thrombin injection therapy. Large pseudoaneurysms, wide-necked morphology, high-flow characteristics, and vessel wall integrity influence the likelihood of thrombus formation and pseudoaneurysm closure [7]. Feld et al. reported that thrombin injection therapy is likely to fail when residual blood flow persists within the pseudoaneurysm. In this case, there is no available ultrasound data at the time of thrombin injection to confirm this; however, it is highly probable that the treatment was unsuccessful due to the persistence of blood flow within the pseudoaneurysm [8]. Additionally, the failure of thrombin injection may not be immediately apparent, as some pseudoaneurysms initially appear stable but subsequently enlarge, as observed in our case. This highlights the necessity of routine postprocedural imaging to confirm successful thrombosis and identify cases requiring further intervention.

Surgical repair remains the gold standard of definitive treatment when thrombin injection fails. Open surgical repair allows the direct identification of arterial defects and the quality of the closure, as well as the prevention of complications such as rupture, distal embolization, or persistent bleeding [9]. Minimally invasive options are increasingly being used; however, surgery remains indispensable in cases that are refractory to percutaneous treatment.

## Conclusions

This case involved a 33-year-old patient who developed a femoral artery pseudoaneurysm following endovascular treatment for multiple traumas. Although the interventional radiology team performed a local thrombin injection, the treatment was unsuccessful. The failure was later identified during follow-up, leading to the first consultation with the vascular surgery team. This case highlights the importance of considering early vascular surgical intervention when a pseudoaneurysm is present.
